# A pilot randomized clinical trial of the Skills to Enhance Positivity (STEP) group intervention for young adults with depression: Examining feasibility and acceptability

**DOI:** 10.1371/journal.pone.0350008

**Published:** 2026-06-10

**Authors:** Katherine M. Tezanos, Natalia Macrynikola, Leanna Villareal, Jackson Doerr, Nazaret Suazo, Laura Whiteley, Shirley Yen

**Affiliations:** 1 Department of Psychiatry and Human Behavior, Alpert Medical School of Brown University, Providence, Rhode Island, United States of America; 2 Harvard Medical School, Beth Israel Deaconess Medical Center, Boston, Massachusetts, United States of America; 3 Department of Psychology, Northeastern University, Boston, Massachusetts, United States of America; 4 Department of Psychology and Neuroscience, University of North Carolina at Chapel Hill, Chapel Hill, North Carolina, United States of America; NYU Grossman School of Medicine: New York University School of Medicine, UNITED STATES OF AMERICA

## Abstract

Addressing the pressing concern of rising rates of depression and suicide in young adults, the present study explores the feasibility of adapting the Skills to Enhance Positivity (STEP) intervention as a group-based program in a community mental health setting in the United States. STEP is an adjunctive intervention designed to enhance positive emotions to reduce suicide risk and alleviate depressive symptoms. Fifty-two participants aged 18–26 (*M* = 21.40, *SD* = 2.00) were randomized to receive group-based STEP or enhanced treatment as usual (ETAU). The group-based STEP format was well-received, with high attendance and positive feedback, demonstrating feasibility and acceptability. While underpowered to detect between-group differences, at post-intervention and follow-up, those in STEP exhibited increases in positive emotions and significant reductions in depression and suicidal ideation. These changes were larger compared to the effect sizes from those in ETAU. These findings underscore the potential of the adapted STEP model to help young adults, warranting further exploration in larger trials. Study is registered on clinicaltrails.gov under ID Number: NCT06621992.

## Introduction

Depression and suicidal thoughts and behaviors (STBs) among adolescents and young adults is a growing issue within the fields of psychology and psychiatry. Young adulthood is an especially important time to intervene, as nearly 75% of lifetime psychiatric disorders emerge by the age of 24 [1]. In recent years, prevalence rates of depression and STBs in this age group have increased, with some studies suggesting that the highest rates of increase are occurring within this age group [2–5]. Recent data suggests that despite these increases and the proliferation of efficacious treatments, treatment utilization continues to lag behind (despite modest increases over the past 10-years), and most young adults do not seek treatment [6]. Accessibility and availability of services [7], as well as young people’s reluctance to engage in individual therapy [8], may contribute to low rates of treatment utilization. In the United States, there are too few trained mental health professionals to meet the rising mental health needs of the youth [9], and this shortage is anticipated to only worsen [10], while depression is projected to become the leading cause of disability in the world by 2030 [11]. New approaches that make efficient use of resources are needed.

Research has demonstrated that group therapy can be a cost-effective, labor-efficient, and beneficial treatment option for individuals with depression and STBs. Several studies have demonstrated its efficacy in reducing both depressive and suicidal symptoms (e.g., [12]). A scoping review highlighted that group interventions for STBs have been shown to decrease several aspects of suicidality, although the literature base is nascent [13]. Additionally, the long term benefits of group therapy have also been observed (see review by [14]). Group therapy has been shown to provide valuable social support and opportunities for participants to share experiences, which can lead to increased self-disclosure and sense of belonging [15].

There are numerous studies on different types of interventions and treatments for depression and STBs, including in-person and mobile interventions that incorporate a range of psychotherapies and group therapy modalities. Most treatments to date focus on predisposing risk factors, targeting individual symptoms of depression, and reducing negative affect, which refers to the extent to which an individual experiences feelings of being upset or unpleasantly aroused [16]. In contrast, few treatments focus on transdiagnostic risk and resilience factors, such as positive affect, the extent to which an individual feels content or pleasantly aroused [16].Indeed, while there are several mindfulness based interventions for depression, many target increasing mindful awareness to the present moment to disengage with negative emotions, and do not focus on increasing positive emotions directly [17]. Low levels of positive emotions have been found to uniquely contribute to suicide risk and depression, independent of other factors in adults and adolescents [18,19]. Two meta-analyses of positive psychology interventions found that these interventions have small to moderate effects in enhancing well-being and decreasing depressive symptoms [20,21]. However, the vast majority of these interventions were directed towards pre-clinical populations, with nearly half of these studies conducted on college populations.

The Skills to Enhance Positivity (STEP) intervention was developed [22,23] to fill these gaps. STEP is an adjunctive treatment that aims to build skills for increasing personal awareness and experience of positive emotions among youth at high risk for suicide and depression. STEP has demonstrated feasibility, acceptability, and initial efficacy in reducing STBs in adolescents [22,23]. The aim of the present study was to examine the preliminary feasibility and acceptability of the STEP program adapted into a group format for young adults with depression and suicidal ideation, as well as to examine STEP’s preliminary efficacy.

## Methods

### Participants

Fifty-three participants, aged 18–26 (*M* = 21.40, *SD* = 2.00), were referred to the study by clinicians from an outpatient young adult behavioral health program in the northeastern United States. One participant dropped out after completing the baseline assessment due to inability to attend group sessions. To be deemed eligible, participants had to endorse a recent history of depression or STBs and had to be English speaking. Participants were also required to have their own mobile device to participate in the texting portion of the study. Individuals who had psychotic disorders or cognitive deficits that prevented them from understanding the intervention were excluded from recruitment, as determined by the referring clinician using clinical judgement.

### Procedures

All study procedures were approved by the Rhode Island Hospital Institutional Review Board (protocol # 0168−16). After informed written consent was collected, participants completed a baseline assessment on STBs, depressive symptoms, positive and negative emotions, as well as clinical and demographic characteristics. Participants were then randomized 2:1 to the STEP intervention or enhanced treatment as usual (ETAU). This unequal randomization schedule was selected to maximize exposure to the treatment condition while maintaining sufficient power for feasibility and acceptability analyses. This method has been effectively used in multiple clinical trials and to good effect (for an informative review on the topic, please see [24]). Randomization was based on urn stratification (i.e., an allocation method ensuring balance across key variables, in our study depression severity, age, and gender), and conducted in REDCap by non-blind researchers. Blinded researchers were involved in participant screening and enrollment. After the active phase (i.e., in-person portion) of treatment, about one month following baseline assessment (hereafter referred to as “post-treatment,”) and four months after baseline (hereafter referred to as “follow-up”), participants completed surveys on clinical outcomes and satisfaction with the intervention. Surveys were administered by a blind-to-treatment condition researcher. Recruitment was conducted from December 6, 2016 to April 3, 2019. Recruitment stopped when the recruitment goal was reached in April 2019. We confirm that all ongoing and related trials for this intervention are registered.

#### Intervention conditions.

For the present study, the STEP intervention (as described elsewhere [22,23]) was adapted to a group format for young adults receiving outpatient treatment for depression. The STEP intervention consisted of four in person group sessions dedicated to psychoeducation on the function of negative and positive emotions as well as teaching skills to enhance attention to positive emotions, including mindfulness, gratitude, and savoring. Group sessions were held in person in a group room at an outpatient mental health clinic, and entry into group occurred on a rolling basis (i.e., no fixed starting point). Participants were not compensated or provided any additional benefit other than receiving the group content for attending sessions to ensure that the experimental condition remained as naturalistic as possible. Randomization and entrance into the group occurred on a rolling basis. Group size ranged from two to six individuals. Groups were led by three different clinical psychology trained post-doctoral level clinicians. Training consisted of reviewing the STEP treatment manual and previous recorded sessions and attendance in the PI’s supervision meetings. Participants in the treatment condition were concurrently enrolled in a 30-day texting component in which they received daily messages that assessed their mood and facilitated practice of that week’s positive emotion skill. Following the initial 30-day component, participants were given the option to extend the text messages for an additional month, either receiving them daily or with reduced frequency of every other day. Participants enrolled in ETAU received healthy lifestyle text messages.

#### Measures.

*Feasibility and Acceptability*. To assess feasibility, we identified the percentage of eligible participants who consented to participation, and the number of sessions and assessments that were completed. Session attendance was recorded by the group facilitator. To assess acceptability, a modified version of the Client Satisfaction Questionnaire (CSQ; [25] was administered to young adult participants at the post-treatment and follow-up timepoints as a self-report form. Modifications were made to examine specific components of the STEP intervention including modality (i.e., group sessions, text messaging) and content (i.e., functions of positive emotions, mindfulness, gratitude, and savoring). Responses were on a 4-point Likert scale with higher scores corresponding to greater satisfaction.

*Positive and negative emotions*. The *Modified Differential Emotions Scale (mDES*; [26]) was used to assess the experience of current positive and negative emotions. The mDES is a 19-item self-report questionnaire that assesses short-term state positive and negative emotions using a 5-point Likert scale (1 = not at all, 5 = extremely). The scale is divided into two subscales: positive emotions (containing 10 items) and negative emotions (containing 9 items). The mDES has strong psychometric properties (reliability alphas (α) = .69–79; [27]). Participants were asked to rate each emotion according to how they were feeling “right now.” The mDES was administered at baseline and at the post-treatment and follow-up assessments.

*Depressive symptoms*. *The Beck Depression Inventory (BDI-II*; [28]) was used to assess current (past two weeks) depressive symptomatology. The BDI-II is a 21-item self-report measure with strong reliability and validity (α = .91 [29]). The BDI-II was administered at baseline and at the post-treatment and follow-up assessments.

*Suicide Ideation*. Suicide ideation was assessed with the *Suicide Ideation Questionnaire* (SIQ; [30]), a 30- item self-report form that assesses severity (i.e., intensity and frequency) of suicidal thoughts. The SIQ is noted to have high internal consistency, test-retest reliability, and construct validity (α = .97 [30]). The SIQ was administered at baseline, post-treatment, and follow-up.

*Mechanisms of treatment*. The following measures were administered at baseline and at the post-treatment and follow-up assessments. *The Gratitude Questionnaire-Six-Item Form* (*GQ-6*; [31]) is a six-item self-report questionnaire used to assess the experience of gratitude in daily life on a 7-point Likert scale (1 = strongly disagree, 7 = strongly agree). A total score is derived by averaging participant responses, with two items reverse scored. The GQ-6 has demonstrated good internal consistency (α = .82; [31]). *The Savoring Beliefs Inven*tory (*SBI*; [32]) is a 24-item self-report scale that assesses an individuals’ perceptions of their ability to derive pleasure from experiences and is comprised of a total score and the following three subscales: 1. Anticipating (upcoming positive events), 2. Savoring (positive moments), and 3. Reminiscing (past positive experiences). For the present study we focused on findings from the Savoring subscale, which has good psychometric properties (α = .86; [32]). The *Five Facet Mindfulness Questionnaire* (*FFMQ;* [33]) is a 39-item self-report questionnaire that assesses five discrete facets of trait mindfulness: Observing (e.g., “I pay attention to sensations, such as the wind in my hair or sun on my face.”), Describing (e.g., “I can easily put my beliefs, opinions, and expectations into words.”), Acting with awareness (e.g., “I find it difficult to stay focused on what’s happening in the present.” Note: This item is reverse scored.), Non-judging of inner experience (e.g., “I tell myself I shouldn’t be feeling the way I’m feeling.” Note: This item is reverse scored.), and Non-reactivity to inner experience (e.g., “I perceive my feelings and emotions without having to react to them.”). Items are rated on a five-point Likert scale ranging from 1 (never or rarely true) to 5 (very often or always true). The FFMQ has good internal consistency (αs = .75−.91; [33]). In this study, we focused on the following three subscales: Awareness, Non-judging, and Non-reactivity.

*Secondary clinical outcome*. Hopelessness was assessed at the baseline, post-treatment, and follow-up assessments. Hopelessness was assessed using the Beck Hopelessness Scale (BHS; [34]). The BHS is a 20-item instrument containing 20 true-false statements. It has high internal reliability across diverse clinical and nonclinical populations (αs = .87− .93). A score in the range of 9–14 indicates moderate hopelessness, while a score of 15–20 corresponds to severe hopelessness.

*Demographic characteristics*. At baseline, demographic characteristics such as age, sex, race, ethnicity, and sexual orientation status were assessed using a self-report questionnaire.

### Data analysis plan

We first conducted independent samples *t*-tests (for continuous variables) and chi-square analyses (for categorical variables) to determine whether there were significant differences in baseline clinical and demographic characteristics between the STEP and ETAU groups.

Descriptive statistics (means, percentages) were computed to determine feasibility of sessions completed, text messaging participation rates, and follow-up rates. Text messaging participation was operationalized as daily rate of providing an appropriate reply to any prompt, which ensured that a skill was delivered for that day. Means and standard deviations for satisfaction items (i.e., modified CSQ) were calculated as a measure of acceptability, with mean satisfaction ratings of 3 or higher (per each item) indicating good acceptability.

While the study was underpowered to compare clinical outcomes between groups, within-group comparisons were made to estimate treatment effect sizes. To do this, paired samples *t*-tests were run to assess changes in treatment targets and secondary clinical outcomes from baseline to post-treatment and baseline to follow-up.

## Results

Complete demographics of the sample are presented in Table 1. Participants were 53 young adults aged 18–26 (*M* = 21.40, *SD* = 2.00). The sample was comprised primarily of young adults assigned female at birth (*n* = 36; 69%). In terms of gender identity, 34 (65%) identified as female. The majority of the sample reported being single, never married (*n* = 49; 94%) and 22 (41%) identified their sexual orientation as lesbian, gay, bisexual, or endorsed another orientation not specified in the questionnaire. There were no significant clinical or demographic differences between groups at baseline.

**Table 1:** Baseline demographic and clinical characteristics.

Variable	STEP (n = 35)	TAU (n = 17)	Total Sample (n = 52)	Test	df	*p*
	*N(%)*	*N(%))*	*N(%)*			
Age (M, SD)	21.41, ± 2.03	21.38, ± 2.00	21.40, ± 2.00	*t* = −0.06	48	.95
Sex assigned at Birth				*X*^*2*^ *= 0.92*	2	.63
Male	9 (26%)	6 (35%)	15 (29%)			
Female	25 (71%)	11 (65%)	36 (69%)			
Gender				*X*^*2*^ *= 0.54*	3	.76
Male	11 (31%)	6 (35%)	17 (33%)			
Female	23 (66%)	11 (65%)	34 (65%)			
Other	1 (3%)	0 (0%)	1 (2%)			
Race				*X*^*2*^ *= 0.90*	2	.34
White	27 (77%)	11 (64%)	38 (73%)			
Black	3 (8%)	1 (6%)	4 (7%)			
Asian	2 (6%)	1 (6%)	3 (6%)			
Other	1 (3%)	2 (12%)	3 (6%)			
Multi Racial	2 (6%)	2 (12%)	4 (7%)			
Ethnicity				*X*^*2*^ *= 0.09*	1	.75
Hispanic/Latino	5 (14%)	3 (18%)	8 (15%)			
Sexual Orientation				*X*^*2*^ *= 0.12*	3	.99
Heterosexual	20 (57%)	10 (59%)	30 (58%)			
Homosexual	4 (11%)	2 (12%)	6 (11%)			
Bisexual	8 (23%)	4 (23%)	12 (23%)			
Other	3 (9%)	1 (6%)	4 (8%)			
mDES Positive M(SD)	1.85 (.61)	1.86 (0.52)	1.85 (.58)	*t* = 0.11	50	.91
mDES Negative M(SD)	2.11 (.81)	2.03 (0.84)	2.09 (.81)	*t* = −0.36	50	.72
GQ6 M(SD)	28.43 (5.70)	26.82 (4.76)	27.90 (5.42)	*t* = −1.00	50	.32
Mindfulness M(SD)						
NonReact	17.26 (5.57)	17.00 (5.27)	17.17 (5.43)	*t* = −0.16	50	.87
Awareness	19.43 (6.18)	19.59 (4.89)	19.48 (5.74)	*t* = 0.09	50	.93
NonJudgement	18.51 (7.73)	19.00 (7.53)	18.67 (7.59)	*t* = 0.21	50	.83
SBI M(SD)	3.77 (.82)	3.89 (1.05)	3.81 (.89)	*t* = 0.48	50	.64
BDI M(SD)	27.66 (11.85)	30.36 (8.60)	28.53 (10.87)	*t* = 0.76	41	.45
SIQ M(SD)	50.40 (30.37)	61.12 (30.93)	53.90 (30.67)	*t* = 1.19	50	.24
BHS M(SD)	9.40 (4.67)	11.29 (4.77)	10.01 (4.74)	*t* = 1.36	50	.18

Notes: mDES = Modified Differential Emotions Scale. GQ6 = The Gratitude Questionnaire. SBI = The Savoring Beliefs Inventory. BDI = Beck Depression Inventory. SIQ = Suicide Ideation Questionnaire. BHS = Beck Hopelessness Scale.

### Feasibility

**Fig 1:**
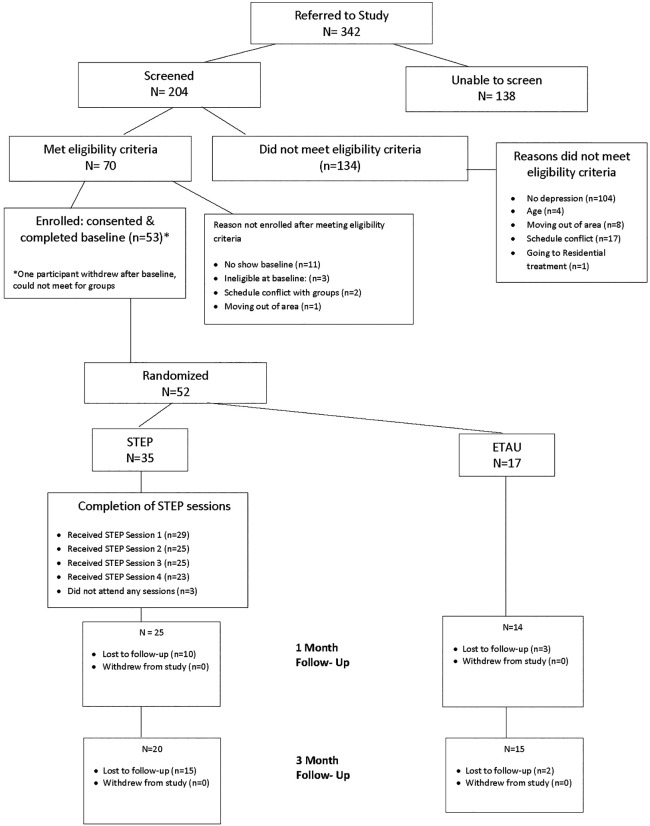
CONSORT flow diagram for trial participants recruited from December 6, 2016 to April 3, 2019.

Eighty-three young adults were screened, and 53 were enrolled in the study. One participant withdrew before being randomized due to anticipated schedule conflict with group times should they be randomized to STEP. Please see Fig 1 for details.

Based on this rate, recruitment and randomization were deemed acceptable. A 2:1 randomization procedure was used in order to maximize exposure to the treatment and ensure sufficient membership in groups. Therefore, 35 participants were randomized to STEP and 17 participants to ETAU.

Thirty-nine participants (75%) completed both baseline and post-treatment assessments, and 35 (67%) completed all three study assessments (i.e., baseline, post-treatment, and follow-up). There were no clinical, treatment group, or demographic characteristics that differentiated those who completed all three follow-ups from those who did not. Due to unexpected personnel turnover, the CSQ was administered to only 51% of individuals assigned to STEP.

For the in-person group component of STEP, out of the 35 young adults randomized to the program, 29 (83%) completed the first session, 25 (71%) completed the second session, 25 (71%) completed the third session, and 23 (66%) completed the fourth session. Over half (*n* = 19, 54%) completed all four STEP group sessions.

The remote skills delivery component of STEP was also feasible. Out of the 35 young adults randomized to the program, during the active phase, participants responded to an average of 21 (75%) daily text prompts. Additionally, 28 (80%) of the STEP participants opted for the text messaging extension. Of those who opted for the extension, 19 (68%) selected to extend messages daily, while 9 (32%) selected the tapered delivery.

### Acceptability

This group adaptation of STEP had high acceptability ratings at the post-treatment and follow-up timepoints. The majority of participants for whom data was obtained rated STEP in the “good” to “excellent” range (Table 2), and rated learning about the function of positive emotions as “somewhat” to “very much” important in their treatment. Participants rated the text messaging component as being as helpful as the group component at post-treatment and slightly more helpful at follow-up. With regard to the specific content of the intervention, participants rated the mindfulness skills as most helpful, with the majority reporting that the skills helped “somewhat” to “a great deal.” Participant ratings are depicted in Table 2.

**Table 2:** Young Adult Satisfaction with STEP – Responses from the CSQ (N = 18).

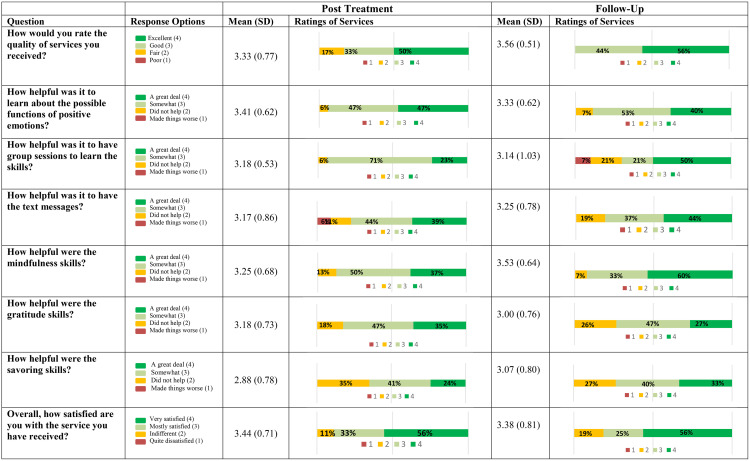

### Preliminary clinical outcomes

*Positive and negative emotions*. For STEP participants, there was a significant within-group increase in positive emotions from baseline to post-treatment and from baseline to follow-up (Table 3). For ETAU participants, only the change from baseline to follow-up was significant. For those in STEP, there was a significant decrease in negative emotions from baseline to follow-up, but not to post-treatment. For those in ETAU, there was no significant change in negative emotions at post-treatment or follow-up.

**Table 3:** Within-group changes in treatment targets from baseline to post-treatment and follow-up timepoints.

	Baseline	Post-Tx	Test				Follow-up	Test			
	*M*(*SD*)	*M*(*SD*)	*t*	*df*	*p*	*d*	*M* (*SD*)	*t*	df	*p*	*d*
mDES Pos	1.85 (0.61)	2.20 (0.80)	2.59	25	**.02**	.51	2.48 (0.84)	5.12	20	**<.001**	1.12
mDES Neg	2.11 (0.81)	1.90 (0.68)	1.98	24	.06	.40	1.88 (0.71)	3.08	20	**<.01**	.67
GC6	28.43 (5.70)	31.40 (5.85)	−2.45	24	**.02**	−.49	31.40 (4.87)	−1.90	19	.07	−.42
Mindfulness											
NonReact	17.26 (5.57)	18.32 (4.06)	−2.21	24	**.04**	−.43	19.55 (3.93)	−2.45	19	**.02**	−.55
Awareness	19.43 (6.18)	19.64 (7.04)	0.03	24	.97	.01	20.15 (6.09)	−0.08	19	.94	−.02
NonJudge	18.51 (7.73)	18.48 (6.70)	−0.69	24	.49	−.14	20.65 (7.60)	−1.87	19	.07	−.42
SBI	3.77 (.82)	4.21 (.88)	−3.55	24	**<.01**	−.71	4.44 (.89)	−3.66	19	**<.01**	−.82
BDI	27.66 (11.85)	22.12 (13.41)	3.78	21	**<.001**	.81	20.25 (12.62)	3.20	18	**<.01**	.73
SIQ	50.40 (30.37)	50.12 (33.94)	0.67	24	.52	.13	42.30 (30.77)	2.58	19	**<.01**	.58
BHS	9.40 (4.67)	8.12 (5.03)	2.71	24	**<.01**	.54	7.00 (5.11)	2.75	24	**<.01**	.54
**ETAU**											
	**Baseline**	**Post-Tx**	**Test**				**Follow-up**	**Test**			
	*M*(*SD*)	*M*(*SD*)	*t*	*df*	*p*	*d*	*M*(*SD*)	*t*	*df*	*p*	*d*
mDES Pos	1.86 (0.52)	2.33 (0.91)	−1.76	12	.10	−.49	2.41 (0.68)	−2.39	13	**.04**	−.60
mDES Neg	2.03 (0.84)	2.02 (0.83)	0.52	13	.61	.14	1.87 (0.68)	0.91	13	.38	.24
GC6	26.82 (4.76)	29.27 (6.81)	−1.69	13	.12	−.45	30.47 (4.93)	−2.53	13	**.03**	−.68
Mindfulness											
NonReact	17.00 (5.27)	19.33 (4.76)	−2.14	13	.05	−.57	18.13 (4.85)	−1.29	13	.22	−.35
Awareness	19.59 (4.89)	20.13 (5.87)	−1.06	13	.31	−.28	21.33 (7.41)	−1.55	13	.15	−.41
NonJudge	19.00 (7.53)	21.47(7.74)	−1.84	13	.09	−.48	23.53 (10.23)	−1.78	13	.10	−.48
SBI	3.89 (1.05)	4.23 (1.10)	−1.34	13	.20	−.35	4.33 (1.06)	−0.75	13	.47	−.20
BDI	30.36 (8.60)	21.00 (10.89)	2.26	11	.05	.65	20.29 (11.42)	2.66	11	**.02**	.77
SIQ	61.12 (30.93)	58.64 (33.17)	1.59	13	.07	.42	56.86 (43.56)	1.39	13	.09	.37
BHS	11.29 (4.77)	8.36 (5.09)	1.62	13	.07	.43	8.21 (5.47)	1.27	13	.11	.34

Notes: mDES = Modified Differential Emotions Scale. GQ6 = The Gratitude Questionnaire. SBI = The Savoring Beliefs Inventory. BDI = Beck Depression Inventory. SIQ = Suicide Ideation Questionnaire. BHS = Beck Hopelessness Scale.

*Depressive symptoms*. Those in the STEP condition reported a significant decrease in depressive symptoms from baseline to post-treatment and from baseline to follow-up (Table 3). Those in ETAU experienced a marginally significant decrease in depression symptoms from baseline to post-treatment, and a significant decrease from baseline to follow-up.

*Suicidal ideation*. Suicidal ideation decreased significantly for those in STEP from baseline to follow-up, though there was not a significant decrease from baseline to post-treatment (Table 3). There was no significant decrease in suicidal ideation for those in ETAU at post-treatment or follow-up.

*Mechanisms of treatment*. The hypothesized mechanisms of mindfulness, gratitude, and savoring showed small to medium effects for those in the STEP condition. There were significant increases in gratitude and savoring from baseline to post-treatment but not from baseline to follow-up, and there was a significant increase in the non-reactivity component of mindfulness from baseline to both post-treatment and follow-up timepoints (Table 3). For those in ETAU, there were no significant changes in gratitude, savoring, or mindfulness at any timepoints except one: Gratitude increased from baseline to follow-up.

*Secondary clinical outcome*. There was a significant decrease in hopelessness for those in the STEP condition at both the post-treatment and follow-up assessment. For those in ETAU there was no significant change in hopelessness at post-treatment or the follow-up (Table 3).

## Discussion

This pilot randomized controlled trial assessed the feasibility, acceptability, and preliminary efficacy of the Skills to Enhance Positivity (STEP) program adapted as a group intervention for young adults with depression in a community mental health setting. The study yielded three key findings: 1) the adapted STEP intervention was both feasible and acceptable for young adults with depression and STBs; 2) the supplemental text messages (to extend the reach of the intervention during and beyond active treatment) were well received; and 3) the proposed positive emotion mechanisms of the intervention (i.e., gratitude, mindfulness, and savoring) and target symptoms (depression and STB outcomes) were largely modified in the anticipated directions and more so for the intervention group. We elaborate on each key finding below.

First, the adapted STEP group intervention was both feasible and acceptable to administer in a group setting. With respect to feasibility, attendance rates were relatively high for the in-person group component of STEP, with attendance rates ranging from 66–83% across sessions. With respect to acceptability, STEP received positive acceptability ratings at post-treatment and follow-up timepoints, with participants finding the psychoeducation on the function of positive emotions and the text messaging component to be particularly important to their treatment (Table 2). Of participants for whom we had data, the majority (93%) rated their overall experience with the intervention as “good” to “excellent.” These ratings are in line with qualitative feedback received as part of the study. Participants offered suggestions on how to improve the intervention, with several participants suggesting that more group sessions would have been helpful to further consolidate skill acquisition. Incorporating direct feedback from participants into future STEP implementations is critical to enhancing STEP's feasibility and acceptability.

Second, the remote delivery phase was also deemed feasible, with high (75%) daily engagement. It was also found to be acceptable, with 83% reporting that the text messages were “somewhat” to a “great deal” helpful. Additionally, a large percentage of the sample (80%) opted into the extension phase. Although elaborating extensively on qualitative aspects of the study is beyond the scope of the present paper, one participant shared that, “Text messages were helpful and made me feel like I wasn’t alone. It was a great reminder to stay strong through hard times.” Several participants also suggested that a longer duration of the text messaging component would have been helpful for skill practice and generalization.

It is promising that the group and remote delivery phase of the intervention were both acceptable and feasible, as there is a need for interventions that can reach people with limited access to care. Individuals with depression and STBs often have low engagement rates with treatment due to a number of barriers including access to care, cost, lack of available providers, and personal beliefs about individual treatment (e.g., [35]). Group and remote delivery interventions may be able to close the gap for some young adults who experience these barriers to treatment. This is especially important for individuals from historically marginalized racial and ethnic backgrounds for whom treatment engagement is often lower [36]. Group delivery of interventions not only imparts the targeted intervention material but also yields additional benefits for participants, including increased perceptions of peer validation and social support, normalization of shared experiences, increased motivation to engage in treatment, as well as the further development of crucial social skills [15]. Indeed, research shows that group characteristics, such as cohesion, can significantly impact treatment outcomes for groups targeting depression and other psychiatric conditions [37]. While, the present study did not assess group characteristics, future studies are encouraged to examine how group characteristics impact treatment outcomes. Beyond these therapeutic benefits, group delivery is also cost-effective and maximizes limited resources, including clinicians’ time (as long as the group contains at least one more member than the number of clinicians running the group), while providing a wider reach of treatment [38].

Finally, while this study was underpowered to test for differences *between* the STEP and ETAU groups, within-group comparisons and effect sizes suggest that the intervention did modify intended targets. Specifically, there were small to moderate effects in reducing suicidal ideation and depressive symptoms, and in increasing positive emotions, gratitude, savoring, and mindfulness in the STEP group. Notably, changes within the STEP condition were generally larger compared to the effect sizes observed in ETAU. Here we briefly note that relative to those in STEP, participants in ETAU demonstrated fewer and less consistent changes over time, with some improvements in depressive symptoms and positive emotions emerging at follow-up, but no significant changes observed in negative emotions, suicidal ideation, hopelessness, or most hypothesized mechanisms of change. Low levels of positive emotions have been shown to be a stronger prospective predictor of suicide risk than negative affect (e.g., [19]), and results from this study suggest that strengthening skills that increase positive emotions may help reduce suicide risk in young adults. In addition, savoring and gratitude are two factors associated with psychological well-being and may act as buffers against depression. Indeed, recent meta-analysis demonstrated that higher levels of gratitude have been linked to lower levels of depression [39]. Thus, interventions that target gratitude, savoring, and potentially other positive psychology constructs may buffer against the symptoms of depression.

### Limitations

Findings should be considered alongside the study’s limitations. First, due to unexpected personnel changes, the CSQ (i.e., measure of client satisfaction), which was administered as a paper-based self-report survey following the participant's last group attendance, was only administered to just over half of the participants in the STEP condition. This means that self-reported satisfaction with the intervention is not as generalizable as we would have preferred. However, there were no clinical, demographic, or engagement related differences between those who did and did not complete the CSQ. Those who did complete the CSQ rated the intervention highly. Future researchers may seek to fully digitize administration of client satisfaction surveys to ensure completion. Relatedly, attendance in group sessions was not as high as we had planned and follow-up data was provided by just under 60% of the STEP condition. These retention rates are similar to what is found in other intervention studies with younger populations (see [40]), and likely reflect the barriers that commonly affect sustained engagement in treatment. These findings underscore the importance of incorporating flexible delivery models when implementing group-based interventions with youth. Third, the sample was small and not adequately powered to observe between-group differences; as such, we cannot fully speak to the efficacy of the treatment; however, preliminary findings were promising. Somewhat related to this point, preliminary efficacy of the treatment was assessed over a 4-month follow-up, a longer follow-up period would help substantiate whether treatment gains are robust and long-lasting. It is important to note that pilot studies are intended to only provide information on feasibility, acceptability, and initial effects on outcomes, and the preliminary data from this study is promising in terms of STEP’s feasibility, acceptability, and potential efficacy in modifying the targets of the intervention. Finally, because the majority of the sample identified as female, the generalizability of findings to males is limited. This gender composition is consistent with other clinical trials, which also tend to overrepresent women [41]. Notably, one study suggests that men may have a slight preference for group-based interventions compared to women [41], highlighting the importance of intentionally recruiting more male participants in future research to better evaluate the broader applicability of this intervention.

### Conclusions and lessons learned

Findings from this study demonstrate that STEP – a brief, adjunctive group intervention with in-person and remote text messaging components to teach skills for enhancing positive emotions and experiences – can be effectively administered to young adults with depression and STBs in community mental health settings. The demonstrated feasibility of delivering STEP in community mental health settings highlights the potential for scalability while its preliminary efficacy suggests that the intervention has the potential to increase positive emotions, a transdiagnostic mechanism of risk, and to reduce suicidal ideation, depression, and hopelessness. Additionally, several key insights emerged from this study that can inform future adaptations and implementation of STEP. First, flexibility in delivery, including remote components, appears to be critical for engagement, particularly for young adults who face logistical barriers to attending in-person sessions. Indeed, the daily text messages prompting mood monitoring and skill practice were especially well received and illustrate the promise of these types of interventions to extend the reach of treatment beyond in-person settings. Future iterations of STEP may do well to increase the duration and perhaps intensity of the mobile mood monitoring portion of the study and offer group content remotely, which may be particularly important for individuals who struggle with access to care. This is especially true following the COVID −19 pandemic in which engagement increased following the widespread implementation of telehealth services and missed appointments decreased [41,42]. Additionally, while participants found the focus on increasing positive emotions beneficial, some also expressed a need for additional support in managing acute distress, suggesting that integrating complementary coping strategies may enhance the intervention’s impact. To replicate and assess effectiveness of this adaptation of STEP, a larger clinical trial will be necessary.
